# Comparative effectiveness of outdoor and exercise interventions for myopia control in children and adolescents: a systematic review and network meta-analysis of randomized clinical trials

**DOI:** 10.3389/fpubh.2026.1833405

**Published:** 2026-07-09

**Authors:** Xiaoquan Weng, Yang Yang, Shuo Zhou, Lingyan Yuan

**Affiliations:** School of Physical Education, Shanghai Normal University, Shanghai, China

**Keywords:** adolescents, axial length, children, exercise, myopia, network meta-analysis, spherical equivalent

## Abstract

**Objective:**

To compare and rank the effects of different sports and outdoor activities on myopia control in children and adolescents using network meta-analysis.

**Methods:**

We systematically searched PubMed, Web of Science, Embase, the Cochrane Library, and major Chinese databases from database inception to Dec 31, 2025, for randomized controlled trials involving school-aged children and adolescents with myopia or at risk of myopia progression. Data were analysed using network meta-analysis in Stata 18.0. Treatment effects were expressed as mean differences (MDs) with 95% CIs, and interventions were ranked using the surface under the cumulative ranking curve (SUCRA).

**Results:**

Sixteen randomized controlled trials involving 9,084 participants were included. Racket sports ranked highest for slowing axial elongation (MD −0.30 mm, 95% CI −0.58 to −0.01; SUCRA 94.9%), followed by increased outdoor time (MD −0.08 mm, 95% CI −0.13 to −0.02; SUCRA 55.7%). For refractive outcomes, visual tracking exercise ranked highest for improving spherical equivalent (MD 0.38 D, 95% CI −0.04 to 0.80; SUCRA 83.7%), followed by racket sports (MD 0.29 D, 95% CI 0.00 to 0.59; SUCRA 77.0%). Subgroup analyses showed that increased outdoor time remained effective in children aged 8.5 years or younger and in interventions lasting more than 24 weeks. Sensitivity analyses supported the robustness of the findings.

**Conclusion:**

Sports and outdoor activities may affect myopia control differently across outcomes. Racket sports showed the most consistent signal for slowing axial elongation, while visual tracking exercise and racket sports ranked higher for spherical equivalent.

**Systematic review registration:**

https://www.crd.york.ac.uk/prospero/display_record.php?ID=CRD420261339612, identifier (CRD420261339612).

## 1 Introduction

In its 2019 World report on vision, the World Health Organization (WHO) identified myopia as a major global public health challenge rather than simply a refractive error. Around 30% of the global population is currently affected, and this figure is expected to reach 50%—approximately 5 billion people—by 2050 (1). High myopia, which is mainly driven by excessive elongation of the eyeball, is associated with a greater risk of sight-threatening complications such as retinal detachment, glaucoma, and cataract (2). In China, myopia has become increasingly common, with earlier onset and greater severity. Prevalence among high school students now exceeds 80% (3), and the rising rate among children aged 6–10 years may further increase the risk of high myopia later in life (4). For this reason, myopia prevention and control in children and adolescents has become a public health priority in China.

The progression of myopia is closely linked to abnormal elongation of the axial length. Prolonged near work may cause eye strain and retinal defocus, which can promote further axial growth (5). Recent studies have also highlighted the role of changes in choroidal thickness and blood flow. Myopia is often associated with a thinner choroid and reduced ocular perfusion, which may limit oxygen supply to the outer eye and make axial elongation more likely (6, 7). In addition, light exposure may influence eye growth through dopamine release. Bright light appears to stimulate dopamine activity and help suppress excessive axial elongation, whereas dim indoor lighting may weaken this protective effect (8, 9).

These findings have drawn attention to the potential role of outdoor activity and exercise in myopia prevention and control. Outdoor exposure may protect against myopia mainly through higher light intensity, increased viewing distance, and reduced continuous near work. Studies have shown that spending about 2 h per day in bright outdoor environments may reduce the onset of myopia and slow ocular growth (8, 9). Exercise may also provide additional benefits, but its effects are unlikely to depend solely on the total amount of activity. Different activities impose different visual and physiological demands. For example, racket sports such as badminton and table tennis require continuous tracking of fast-moving targets, frequent changes in fixation distance, and rapid eye–hand coordination, whereas aerobic outdoor activities may act more through light exposure, physical movement, and improved ocular circulation (7, 10–11).

Recent evidence suggests that physical activity should not be treated as a single homogeneous exposure. Different activity patterns, such as racket sports, ball games, swimming, and mixed recreational activities, may have different associations with myopia, and these associations may vary by educational stage and sex. In particular, activity patterns dominated by ball games have been associated with a lower risk of myopia among middle school students, suggesting that the visual-health effects of physical activity may depend on activity type, visual tracking demands, movement characteristics, and developmental stage rather than on activity participation alone (12).

However, although previous reviews and meta-analyses have examined outdoor time, physical activity, or exercise interventions for myopia prevention and control, several gaps remain (13–16). First, many reviews have treated activity-related interventions as broad categories, making it difficult to distinguish the effects of outdoor exposure, structured outdoor exercise, racket sports, visual tracking exercise, and general physical activity. Second, some previous syntheses relied partly on visual acuity outcomes, which are less able to distinguish structural progression from refractive change. Third, the comparative effects of different activity-based interventions have not been fully examined using both AL and SE within the same randomized evidence framework. Therefore, this study used a network meta-analysis of randomized controlled trials to compare and rank different outdoor and exercise interventions for myopia prevention and control in children and adolescents, using AL and SE as the main outcomes.

## 2 Data sources and methods

### 2.1 Protocol and registration

This systematic review and network meta-analysis was registered in PROSPERO (CRD420261339612). The study was conducted and reported in accordance with the PRISMA 2020 statement and the PRISMA extension for network meta-analyses.

### 2.2 Literature search

We systematically searched PubMed, Web of Science, Embase, and the Cochrane Library, together with the Chinese databases CNKI, Wanfang, VIP, and SinoMed. The search covered the period from database inception to Dec 31, 2025, with no language restrictions. We combined controlled vocabulary and free-text terms related to myopia or refractive error, children or adolescents, outdoor activity, outdoor time, exercise, physical activity, and related subtypes, as well as randomized controlled trials. Terms for the main outcomes, including axial length (AL) and spherical equivalent (SE) or refractive error, were also included. Search terms were combined using the Boolean operators AND and OR.

### 2.3 Eligibility criteria

Randomized controlled trials were eligible if they included school-aged children and adolescents, generally 5–18 years of age, with myopia or at risk of myopia progression. As this review focused on prevention and progression control rather than reversal of established myopia, studies were required to report at least one progression-related outcome, such as AL or SE.

We excluded conference abstracts, studies without accessible full text, and trials involving participants with severe comorbidities, such as neurological or immune disorders. We also excluded studies that did not clearly describe the exercise intervention, reported substantial missing or unusable outcome data, or used clearly inappropriate analyses.

### 2.4 Intervention classification

Interventions were classified according to both activity content and environmental exposure. Physical activity was used as an umbrella term referring to bodily movement or exercise-based interventions, regardless of whether they were performed indoors or outdoors. Outdoor exercise referred to structured physical activity performed in an outdoor setting, in which both exercise and outdoor light exposure may contribute to the intervention effect. Increased outdoor time referred to interventions designed primarily to increase exposure to outdoor environments, such as additional outdoor recess, outdoor class time, or outdoor free activity, without requiring a specific exercise intensity or structured exercise programme. Racket sports and visual tracking exercises were classified separately because they involve repeated tracking of moving targets, frequent fixation shifts, and eye–hand coordination. General physical activity referred to exercise or activity programmes that did not clearly emphasize outdoor exposure or specific visual tracking demands. For mixed or combined interventions, classification was based on the dominant intervention component and the main mechanism described by the original trial. When an intervention could fit more than one category, the final classification was determined by discussion between two reviewers.

### 2.5 Study selection and data extraction

After duplicate records were removed using Zotero 7, two reviewers independently screened titles and abstracts, followed by full-text assessment using the same eligibility criteria. Disagreements were resolved through discussion and, when necessary, by consultation with a third reviewer.

Data were extracted using a pilot-tested form. Two reviewers independently extracted and cross-checked all data to ensure accuracy. The extracted information included study characteristics, baseline participant characteristics, details of the intervention and control conditions, outcome data, and key methodological information.

### 2.6 Risk of bias assessment

Two reviewers independently assessed risk of bias using the Cochrane Risk of Bias 2 tool. The assessment covered the randomisation process, deviations from the intended intervention, missing outcome data, outcome measurement, and selective reporting. Any disagreements were resolved through discussion or consultation with a third reviewer.

### 2.7 Network meta-analysis and statistical analysis

The network meta-analysis was conducted in Stata 18 using a consistency model to combine direct and indirect evidence, while accounting for correlations in multiarm trials. AL and SE were analysed as continuous outcomes and were reported as mean differences (MDs) with 95% CIs. Network plots were constructed to show the available direct comparisons and the overall distribution of evidence.

We assessed heterogeneity within the network and examined inconsistency in closed loops using both global and local approaches. Interventions were ranked using the surface under the cumulative ranking curve (SUCRA), and ranking probability plots were generated.

To examine whether the results varied across study characteristics, subgroup analyses were performed according to follow-up duration (≤24 weeks vs. >24 weeks) and mean baseline age (≤8.5 years vs. >8.5 years). The age cut-off of 8.5 years was based on the median of the available study-level mean baseline ages and was used to maintain a sufficient number of studies within each subgroup. This cut-off was not intended to represent the onset of adolescence or puberty. Effect estimates and rankings were reassessed within each subgroup. Sensitivity analyses were then conducted by excluding studies at high risk of bias to evaluate the robustness of the findings and to determine whether the conclusions or rankings changed.

## 3 Results

### 3.1 Study selection

**Figure 1:**
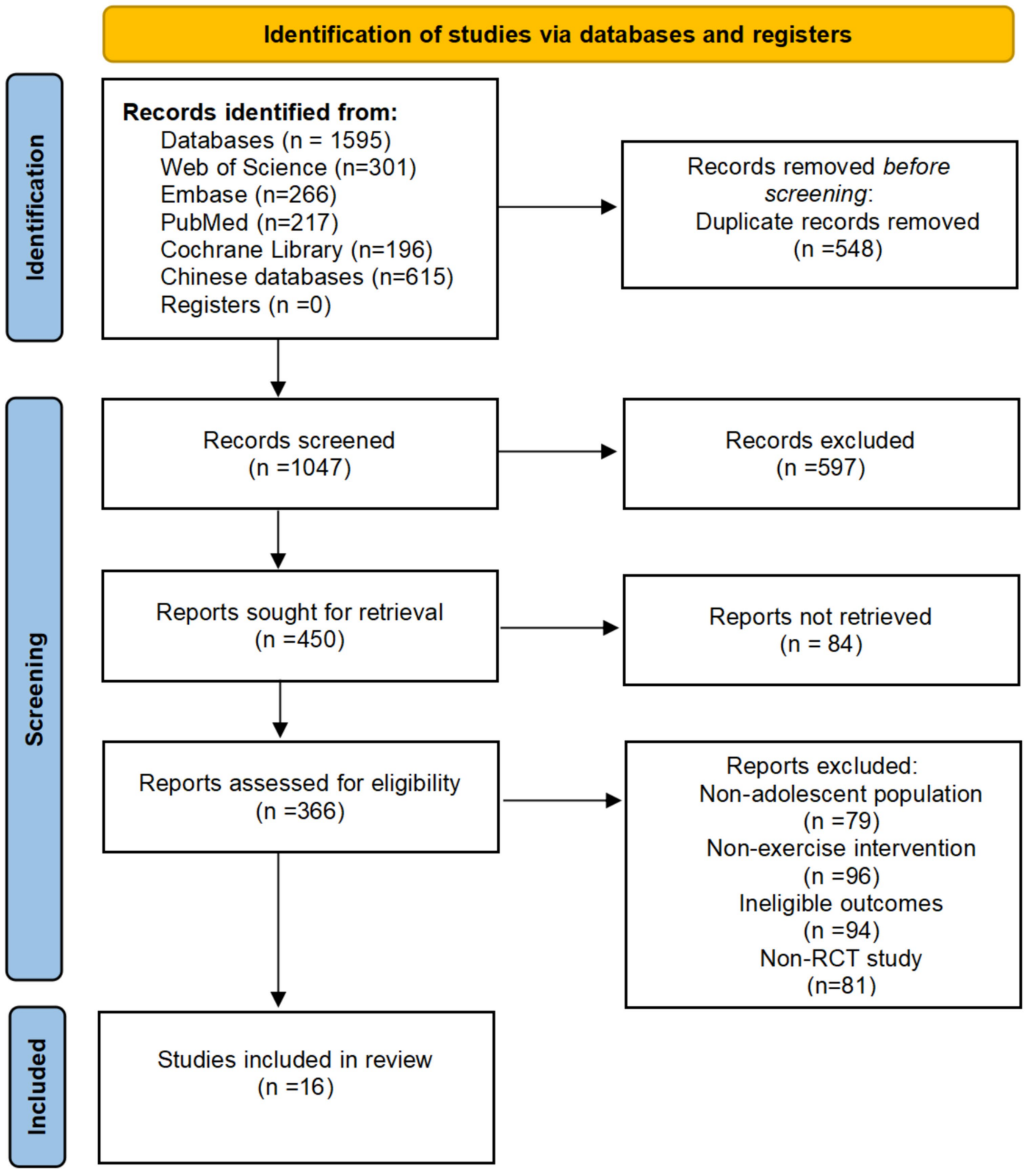
Literature screening flowchart.

A total of 1,595 records were identified through the database search. After screening, 16 randomized controlled trials (RCTs) were included in the network meta-analysis (Figure 1).

### 3.2 Study characteristics and risk of bias

The 16 included RCTs were published between 2015 and 2025 and involved a total of 9,084 participants. Interventions included racket sports, visual tracking exercise, structured outdoor exercise, increased outdoor time, and general physical activity. Control groups generally received usual activities or no regular physical exercise. Intervention frequency ranged from 2 to 15 sessions per week, and study duration ranged from approximately 8 to 156 weeks. Further details are provided in Table 1.

**Table 1:** Basic characteristics of the included literature.

Study ID	Sample size (*n*)	Age (years)	Intervention method	Intervention frequency (times/week)	Intervention cycle (weeks)	Outcome
Zawistowska 2025 (29)	40/62	7–14	A	3	43	AL; SE
Liao 2023 (18)	24/25/26/25	NR	B/C/E	3	52	SE
He 2015 (27)	952/951	6–7	C	5	156	AL; SE
Jin 2015 (30)	1,735/1,316	7–13	C	10	52	AL; SE
Wu 2018 (20)	267/426	5–7	C	15	52	AL; SE
GUO 2019 (31)	157/216	6–8	B	5	52	AL; SE
Zhu 2021 (32)	20/20	NR	E	3	16	SE
Zheng 2025 (33)	91/91	7–9	A	2	52	SE
Zhang 2025 (34)	89/89	8–9	D	3	40	AL; SE
Cao 2023 (35)	15/15/15	NR	A	2	8	SE
Lin 2025 (36)	12/12/12	7–9	A	3	24	AL; SE
Chen 2023 (37)	8/8/8/8	9–11	A/D	3	16	AL; SE
Xia 2024 (38)	20/21	NR	D	3	16	AL; SE
Wang 2023 (39)	60/60	10–11	B	3	10	SE
Li 2018 (40)	357/353/366	NR	C	5	52	AL; SE
Lao 2019 (41)	552/560	NR	C	5	52	AL; SE

NR indicates not reported. Exercise types: A refers to racket sports; B refers to structured outdoor exercise; C refers to increasing outdoor time; D refers to visual tracking sports; E refers to general physical exercise. Outcome indicators: AL is axial length; SE is spherical equivalent.

**Figure 2:**
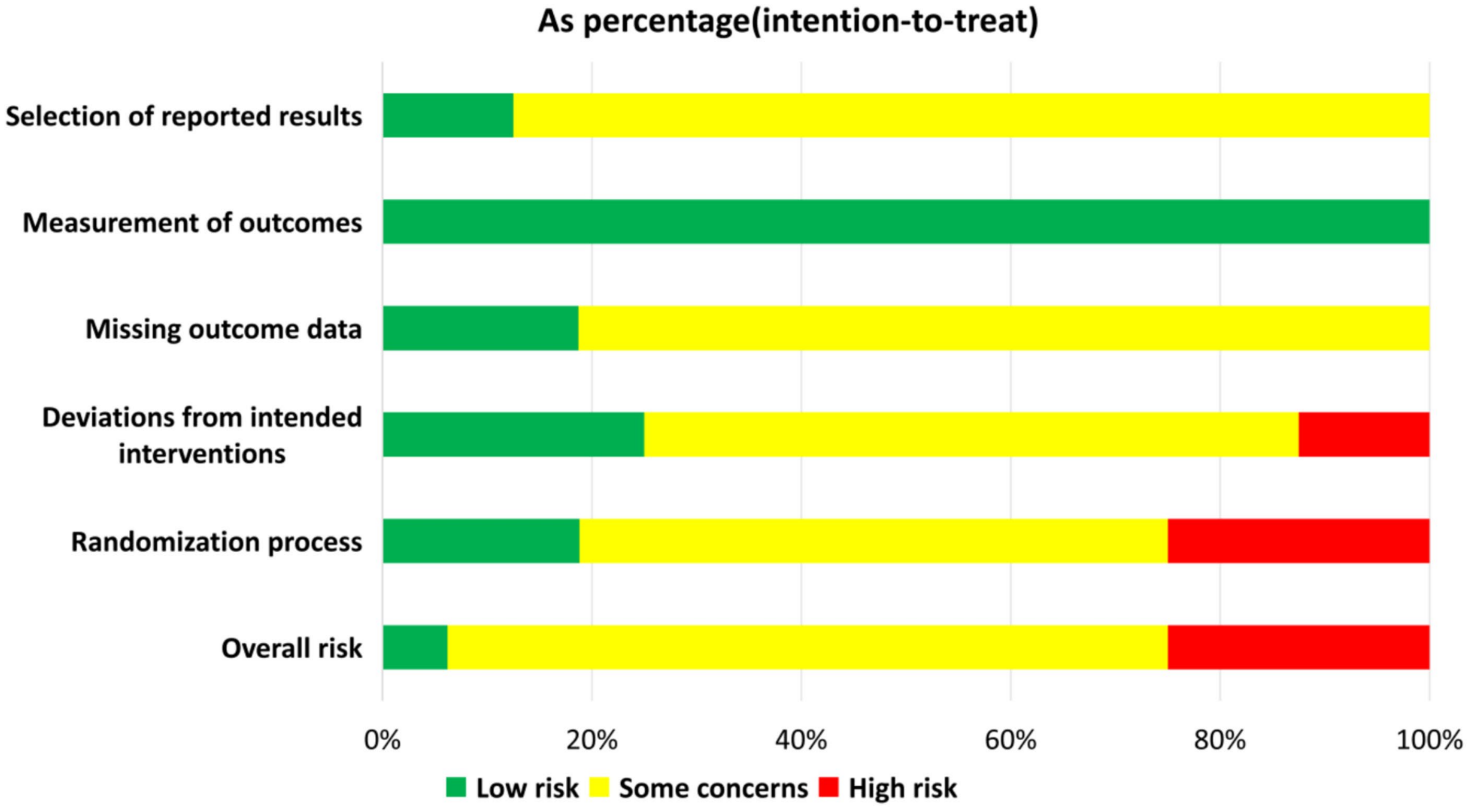
Risk assessment diagram of inclusion bias.

All studies reported the SE change values, among which 11 also reported AL. The RoB 2 assessment showed that the overall risk of bias was mainly “some concerns,” as detailed in Figure 2.

### 3.3 Publication bias

**Figure 3:**
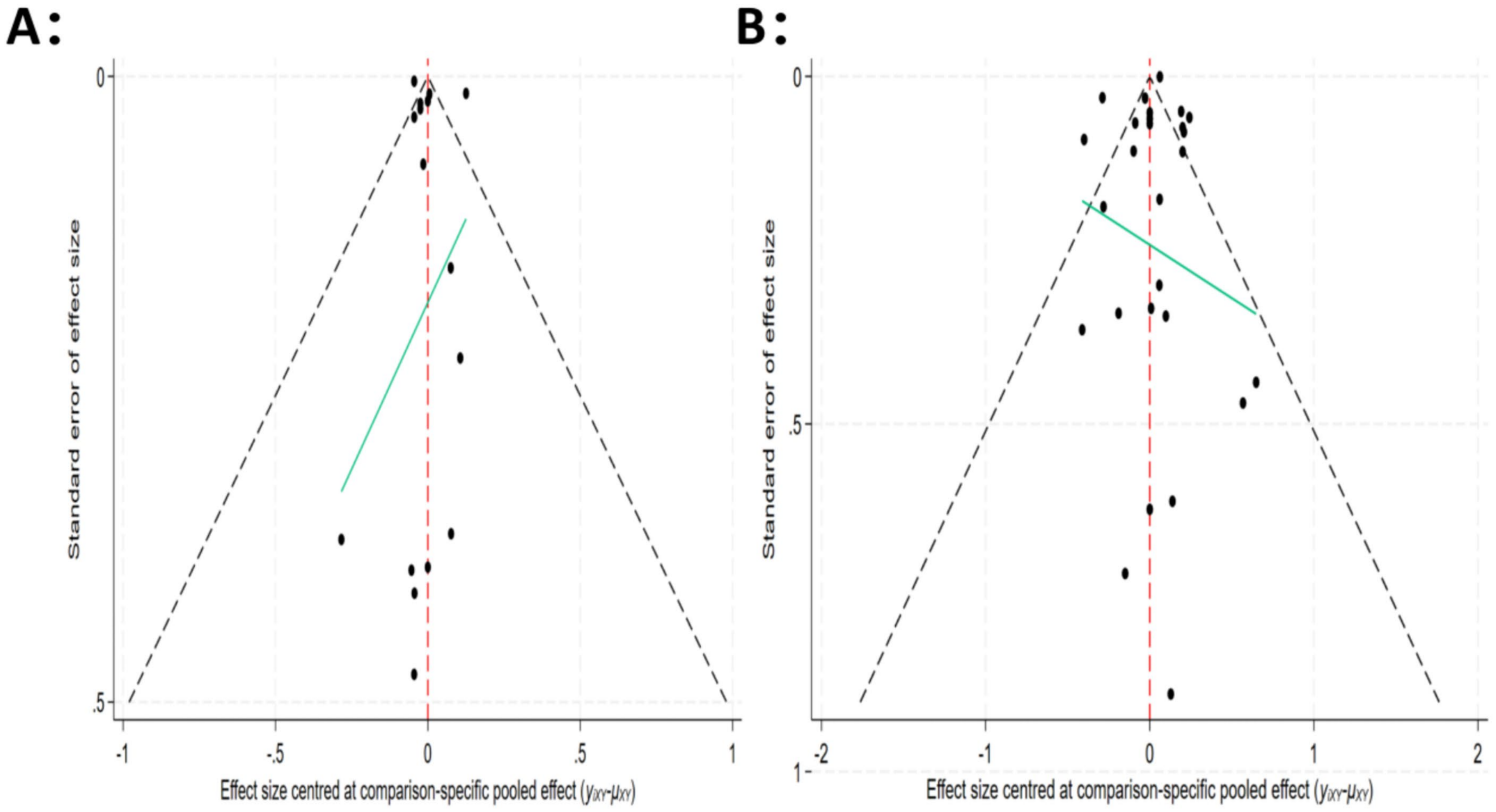
Comparison-adjusted funnel plots: **(A)** is AL; **(B)** is SE.

Comparison-adjusted funnel plots are shown in Figure 3. Most studies were distributed around the line of no effect, and the overall pattern was reasonably symmetrical. However, slight asymmetry was observed, suggesting possible publication bias. Given the limited number of included studies and the structure of the network, this finding should be interpreted cautiously.

### 3.4 Consistency and heterogeneity

Global inconsistency testing showed no significant inconsistency in either network (AL: *p* = 0.9928; SE: *p* = 0.6085). Node-splitting analyses likewise found no significant differences between direct and indirect evidence for key comparisons (all *p* > 0.05), supporting the use of a consistency model. Under the random-effects model, the estimated heterogeneity standard deviation (*τ*) was 0.062 mm for AL and 0.21 D for SE.

## 4 Main findings

### 4.1 Axial length

**Figure 4:**
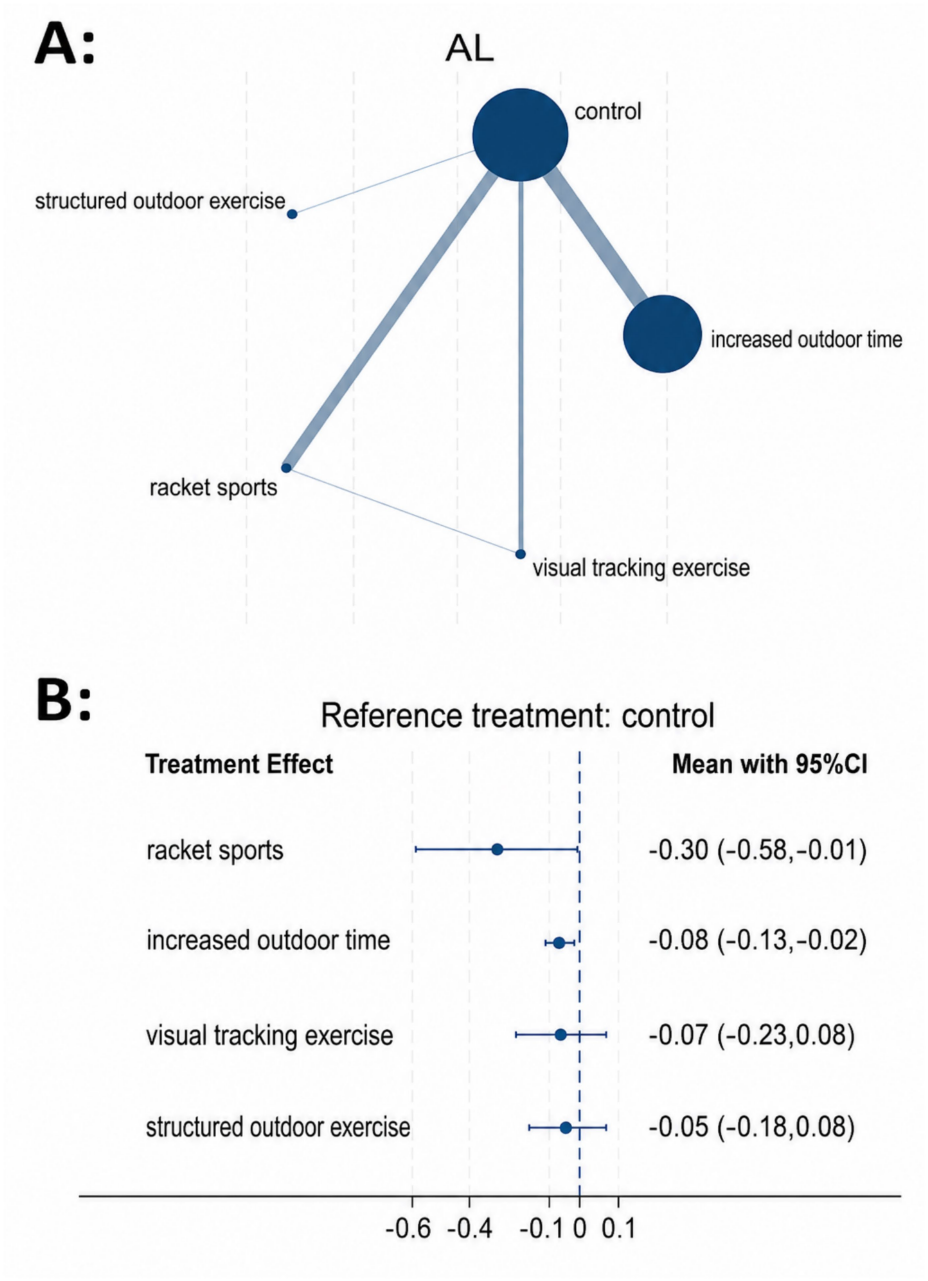
AL network and forest plot. **(A)** Network plot; **(B)** Forest plot.

For AL, the network meta-analysis included five interventions: control, increased outdoor time, structured outdoor exercise, racket sports, and visual tracking exercise. Most direct comparisons were made with the control group, particularly for increased outdoor time and racket sports. The network was well connected, with no disconnected comparisons. Overall, most interventions favored a reduction in axial elongation compared with the control group. Racket sports ranked highest for slowing axial elongation (MD = −0.30 mm, 95% CI −0.58 to −0.01; SUCRA = 94.9%), followed by increased outdoor time (MD = −0.08 mm, 95% CI −0.13 to −0.02; SUCRA = 55.7%; Figure 4).

### 4.2 Spherical equivalent

**Figure 5:**
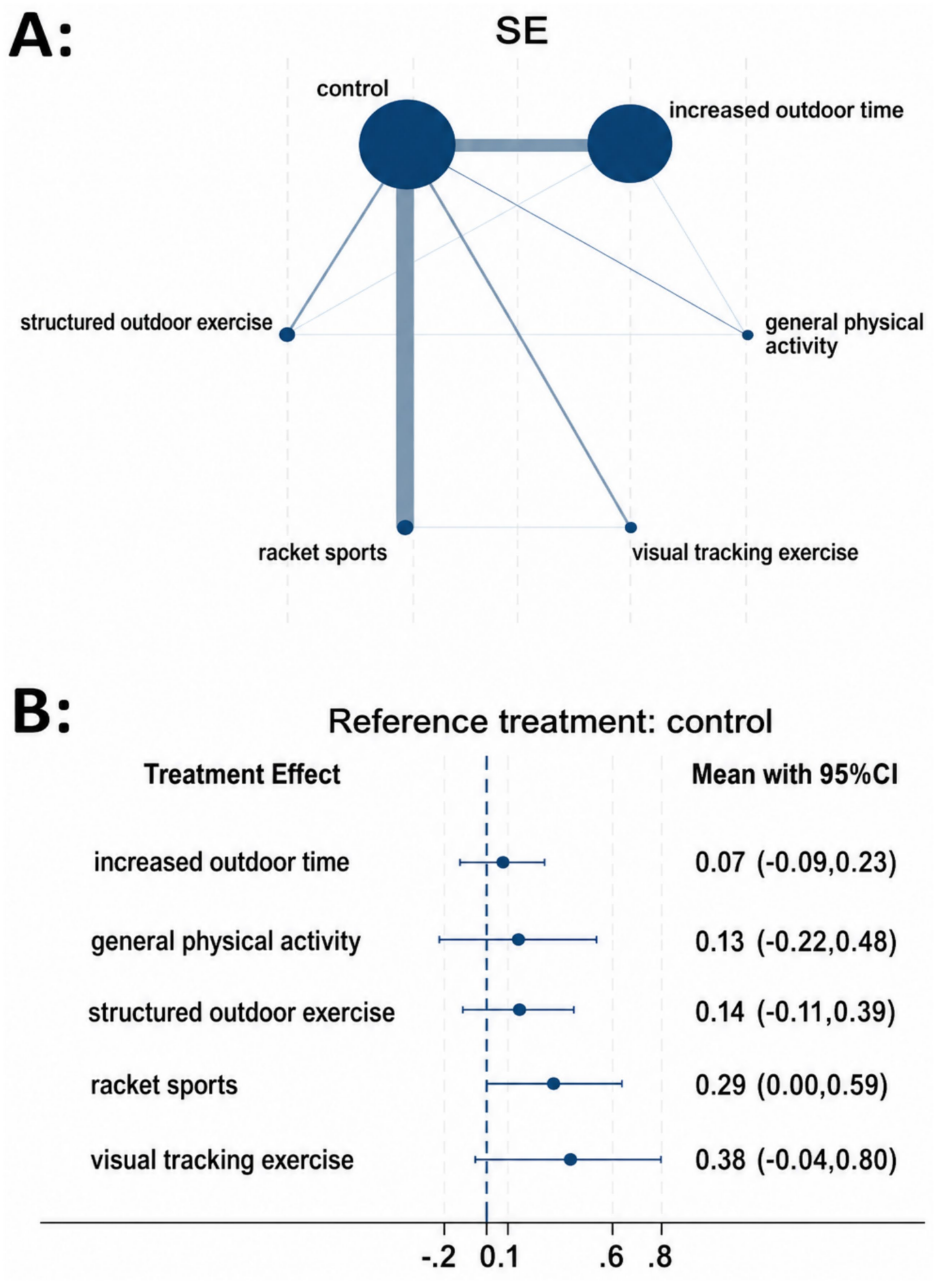
SE network and forest plot. **(A)** Network plot; **(B)** Forest plot.

For SE, the network meta-analysis included six interventions: control, increased outdoor time, structured outdoor exercise, racket sports, visual tracking exercise, and general physical activity. Most direct comparisons were made with the control group. The network was well connected, with no disconnected comparisons. Overall, most interventions favored improvement in SE compared with the control group. Visual tracking exercise ranked highest for improving SE, although the confidence interval crossed the null value (MD = 0.38 D, 95% CI −0.04 to 0.80; SUCRA = 83.7%), followed by racket sports (MD = 0.29 D, 95% CI 0.00 to 0.59; SUCRA = 77.0%; Figure 5).

### 4.3 Subgroup analyses

As shown in Table 2, the effect of increased outdoor time on AL appeared more consistent in children aged 8.5 years or younger and in studies with follow-up longer than 24 weeks. For SE, some interventions showed potential benefit in studies with follow-up of 24 weeks or less. In studies with follow-up longer than 24 weeks, most effects were not statistically significant, although the rankings suggested that visual tracking exercise may still be among the better-performing interventions. Because the subgroup evidence was limited, these findings should be considered exploratory.

**Table 2:** Subgroup analysis results.

Outcome	Intervention	Age ≤8.5 years old	Age >8.5 years old	intervention duration ≤24 weeks	intervention duration >24 weeks
AL (mm)	A	−0.28 (−0.81, 0.25), 78.9	−0.29 (−0.62, 0.03), 93.3	−0.35 (−0.71, 0.00), 95.6%	−0.19 (−0.65, 0.27), 71.7%
	B	−0.05 (−0.19, 0.09), 42.5	–	–	−0.05 (−0.18, 0.08), 44.3%
	C	−0.08 (−0.14, −0.02), 58.5	−0.05 (−0.10, 0.00), 55.0	–	−0.07 (−0.13, −0.02), 59.3%
SE (D)	D	−0.09 (−0.28, 0.10), 55.5	−0.01 (−0.29, 0.27), 34.1	−0.02 (−0.30, 0.26), 30.3%	−0.09 (−0.28, 0.10), 59.3%
	A	0.11 (−0.27, 0.49), 52.4	0.54 (0.13, 0.95), 78.7	0.43 (0.10, 0.76), 65.9%	0.07 (−0.39, 0.53), 48.3%
	B	0.05 (−0.24, 0.34), 43.3	0.32 (0.11, 0.53), 57.3	0.32 (0.11, 0.53), 50.4%	0.04 (−0.26, 0.34), 45.0%
	C	0.09 (−0.09, 0.26), 53.3	−0.17 (−0.29, −0.05), 1.7	–	0.05 (−0.11, 0.21), 49.1%
	D	0.48 (−0.06, 1.02), 91.4	0.27 (−0.31, 0.85), 51.5	0.25 (−0.33, 0.83), 42.0%	0.48 (−0.07, 1.03), 91.8%
	E	−0.01 (−0.38, 0.37), 31.7	0.73 (0.02, 1.44), 86.6	0.73 (0.02, 1.44), 86.0%	−0.02 (−0.41, 0.36), 33.2%

The table shows the results of subgroup analysis, with effect sizes expressed as MD (95%CI) followed by SUCRA (%). A higher SUCRA value indicates a better relative ranking of the intervention within the same subgroup. “–” indicates that the intervention was not included in the subgroup network or there was a lack of mergeable data. Intervention codes: A = racket sports, B = structured outdoor exercise, C = increased outdoor time, D = visual tracking sports, E = general physical exercise.

### 4.4 Sensitivity analyses

After excluding studies at high risk of bias, the network meta-analysis was repeated. The results for both AL and SE remained consistent with the main analysis, and the changes in effect estimates were small, indicating that the findings were robust. For AL, racket sports and increased outdoor time continued to show benefit in slowing axial elongation. For SE, the overall pattern remained stable, although the confidence intervals for some interventions crossed the null value, indicating residual uncertainty (Table 3).

**Table 3:** Sensitivity analysis results.

Interventions	AL (mm)		SE (D)	
	Original data	excluding high risk-of-bias studies	Original data	excluding high risk-of-bias studies
A	−0.30 (−0.58, −0.01)	−0.35 (−0.72, −0.01)	0.29 (0.00, 0.59)	0.27 (−0.03, 0.57)
B	−0.05 (−0.18, 0.08)	–	0.14 (−0.11, 0.38)	0.30 (0.04, 0.55)
C	−0.08 (−0.13, −0.02)	−0.08 (−0.15, −0.02)	0.07 (−0.09, 0.22)	0.08 (−0.06, 0.23)
D	−0.07 (−0.23, 0.09)	−0.02 (−0.32, 0.28)	0.38 (−0.04, 0.80)	0.22 (−0.42, 0.86)
E	–	–	0.11 (−0.20, 0.42)	0.07 (−0.25, 0.40)

A = racket sports, B = structured outdoor exercise, C = increasing outdoor time, D = visual tracking sports, E = general physical exercise. “–” indicates that the intervention was not included in the sensitivity analysis or there was a lack of mergeable data.

## 5 Discussion

### 5.1 Principal findings

This network meta-analysis included 16 randomized controlled trials involving 9,084 children and adolescents and compared different outdoor and exercise interventions for myopia prevention and control. By assessing both axial length (AL) and spherical equivalent (SE), we found that the effects differed across intervention types and outcomes. Racket sports and increased outdoor time showed the most consistent signals of benefit for slowing axial elongation, whereas visual tracking exercise and racket sports ranked higher for SE. These findings suggest that activity-based myopia interventions should not be considered as a single homogeneous category, because different activity patterns may have different associations with myopia and may vary by educational stage and sex (12). Instead, their effects may depend on the activity setting, visual demands, movement characteristics, intervention duration, and age of the target population (11, 12).

### 5.2 Outdoor exposure and physical activity represent different intervention pathways

A key theme emerging from the evidence is the need to distinguish outdoor exposure from physical activity. Increased outdoor time may influence myopia mainly through greater light exposure, longer viewing distance, and reduced continuous near work, even when the activity is not vigorous or highly structured (8, 14). In contrast, physical activity may have additional effects through body movement, ocular circulation, and visual–motor coordination (11). Structured outdoor exercise lies between these two pathways because it combines physical movement with exposure to outdoor light (17, 18).

This distinction is important for interpreting the intervention rankings. The benefit of increased outdoor time for AL may be largely related to light-related and behaviour-related mechanisms, including brighter illumination, more frequent distance viewing, and less sustained near work (8, 9). By contrast, the potential effects of racket sports and visual tracking exercise may be more closely related to dynamic visual demands, such as tracking moving targets, shifting fixation distance, and coordinating eye and body movements (19). Because most included trials did not objectively measure sunlight exposure, light intensity, or time spent under different lighting conditions, sunlight exposure was considered part of the intervention context rather than an independent treatment node in this review (9, 20).

### 5.3 Visually demanding activities may have distinct effects on AL and SE

The results also suggest that visually demanding activities may have different implications for structural and refractive outcomes. For AL, racket sports ranked highest, followed by increased outdoor time, which is consistent with previous evidence suggesting that dynamic visual activities and outdoor exposure may contribute to myopia prevention and control through different pathways (14, 19). Racket sports require repeated changes in fixation, eye movements, rapid visual responses, and eye–hand coordination; these characteristics may help reduce visual strain associated with prolonged near work and may support healthier ocular regulation (19). Their aerobic component may also contribute to ocular blood flow and choroidal responses, which could be relevant to axial elongation (21, 22). Increased outdoor time may act through a different but complementary pathway, mainly involving stronger light exposure, longer viewing distance, and reduced near work (8, 14). Bright outdoor light may stimulate retinal dopamine release, which is thought to help limit excessive eye growth (9, 23).

For SE, visual tracking exercise and racket sports ranked higher than other interventions. Unlike AL, SE reflects both structural eye growth and shorter-term changes in refractive and focusing status, and it may therefore be more sensitive to early changes but more variable over longer follow-up (24). Activities that require children to follow fast or unpredictable targets and frequently shift focus between near and far distances may influence refractive outcomes earlier than they influence structural outcomes (25, 26). These findings suggest that AL and SE should be interpreted as complementary rather than interchangeable outcomes, because AL better reflects structural progression whereas SE provides information on refractive status (11, 24).

### 5.4 Age and duration may shape intervention response

Another theme emerging from the subgroup analyses is that intervention effects may vary by age and duration. Increased outdoor time appeared to be more beneficial in younger children, while older children seemed to respond better to racket sports and visual tracking exercise. This pattern may reflect differences in visual behaviour, academic burden, outdoor time, and participation preferences across school stages (12). Younger children may benefit more from simple increases in outdoor exposure because their visual habits and school routines may be more modifiable, while school-based outdoor programmes have shown benefits in younger children in randomized trials (20, 27). Older children and adolescents, who often face greater near-work demands and reduced free outdoor time, may require more structured and engaging activities to achieve meaningful exposure and adherence (12).

Intervention duration may also matter. Shorter interventions may first influence SE, whereas longer interventions may be needed to produce more stable changes in AL, because AL reflects structural ocular growth and may require longer follow-up to detect sustained change (11, 24). However, these subgroup findings should be interpreted cautiously because the available evidence was limited, and the age cut-off was data-driven rather than based on a clinical definition of adolescence. Future studies should report age-stratified data and test developmentally appropriate intervention strategies across different school stages (12).

### 5.5 Added value compared with previous reviews

Overall, our findings are consistent with existing guidance while providing a clearer comparison across intervention types. The WHO has identified insufficient outdoor exposure and high levels of near work as important contributors to myopia and has promoted increased outdoor time as a cost-effective public health strategy (28). Previous systematic reviews and meta-analyses have also suggested that outdoor time and physical exercise may benefit myopia control in children and adolescents (14–16). However, many previous reviews relied partly on visual acuity outcomes, which can be affected by participant cooperation, testing conditions, and short-term fluctuation (15, 16). By contrast, this study examined both AL and SE, allowing structural progression and refractive status to be considered within the same analytical framework (11). We therefore interpreted stronger evidence mainly from randomized trials and objective outcomes such as AL, while treating findings based on visual acuity, mechanistic explanations, or subgroup rankings as more exploratory.

In addition, this study separated increased outdoor time, structured outdoor exercise, racket sports, visual tracking exercise, and general physical activity within the same comparative framework. This approach allowed a clearer assessment of how different activity modalities may affect myopia-related outcomes and addressed an important limitation of conventional pairwise meta-analysis (13, 16). The findings therefore add to previous reviews by focusing on randomized controlled trials, incorporating both English- and Chinese-language evidence, distinguishing intervention mechanisms more explicitly, and comparing multiple activity-based strategies within one network (13, 14).

### 5.6 Implications for school- and family-based myopia prevention

From a public health perspective, the findings support a more differentiated approach to activity-based myopia prevention. Schools may consider increasing outdoor exposure through recess, outdoor classes, or outdoor physical education, especially for younger children, because school-based trials have shown that increasing outdoor time can reduce myopia onset or progression (20, 27). At the same time, visually engaging activities such as badminton, table tennis, ball games, or structured visual tracking activities may be considered as part of school-based health promotion programmes, particularly when they are feasible, enjoyable, and age-appropriate (12). These findings also have implications for public health education and health promotion. Prevention messages should not only encourage children to “exercise more,” but should also emphasize sufficient outdoor exposure, reduced prolonged near work, and participation in age-appropriate activities with dynamic visual demands.

For families, the findings suggest that myopia prevention should not focus only on reducing screen time or near work, but also on creating regular opportunities for outdoor exposure and visually engaging movement (8, 14). However, the current evidence does not support a single universal intervention for all children, because intervention effects appear to differ by outcome, activity type, age, and intervention duration (11, 12). Intervention choice should therefore consider age, baseline visual status, school workload, access to outdoor space, safety, cost, and adherence. The practical implication of this review is not that one activity should replace all others, but that myopia prevention programmes may be strengthened by combining sufficient outdoor exposure with activities that provide dynamic visual stimulation (12, 14).

### 5.7 Limitations and future directions

Several limitations should be considered. First, few high-quality head-to-head trials directly compared active interventions, and some estimates therefore relied mainly on indirect evidence, which is a common challenge in network meta-analysis when direct comparisons are limited (13, 16). Second, the age-based subgroup analysis should be interpreted cautiously because the cut-off was data-driven rather than based on a clinical definition of adolescence. Most included trials were conducted in primary-school children, and limited age-stratified data prevented a robust adolescence-based subgroup analysis (20, 27). Third, blinding is difficult in exercise-based studies, and many trials were judged to have some risk of bias; publication bias also cannot be excluded. Fourth, cycloplegia was not consistently used for SE measurement in some studies, which may have affected the comparability of refractive outcomes across trials (11). Fifth, most included trials did not objectively measure outdoor light exposure, exercise intensity, or time spent in different activity settings, limiting our ability to separate the independent effects of sunlight exposure from those of physical activity (9, 20).

Future research should move beyond asking whether outdoor or exercise interventions are effective and should instead examine which combinations work best, for whom, and under what conditions. Priority should be given to multicentre head-to-head randomized trials that compare increased outdoor time, structured outdoor exercise, racket sports, visual tracking exercise, and combined strategies (13, 16). Intervention dose, frequency, duration, and intensity should be clearly defined and reported, because current studies vary substantially in intervention frequency and follow-up duration. Future studies should also use objective measures, such as wearable light sensors and activity monitors, to quantify outdoor exposure and physical activity more accurately (9, 20). In addition, cycloplegic refraction, AL, and other ocular biomarkers should be measured consistently to improve comparability across trials (11, 24). Finally, implementation studies are needed to assess feasibility, adherence, equity, and scalability in real school and community settings, especially because school-based outdoor and activity programmes may be important components of public health-oriented myopia prevention (27, 28, 42–44).

## 6 Conclusion

This network meta-analysis of 16 randomized controlled trials involving 9,084 children and adolescents suggests that sports and outdoor activities may affect myopia control differently across outcomes. Racket sports showed the most consistent signal for slowing axial elongation, while visual tracking exercise and racket sports ranked higher for improving spherical equivalent; increased outdoor time appeared particularly beneficial in younger children. These findings may inform school- and family-based myopia prevention, but should be interpreted cautiously because direct comparisons were limited and some estimates were imprecise.
